# Clinical application of CT-based radiomics model in differentiation between laryngeal squamous cell carcinoma and squamous cell hyperplasia

**DOI:** 10.3389/fmed.2023.1337723

**Published:** 2024-01-11

**Authors:** Fengzhi Cui, Osama A. Khodrog, Wei Liu, Jianhua Liu, Qinghai Yuan

**Affiliations:** Department of Radiology, The Second Hospital of Jilin University, Changchun, China

**Keywords:** laryngeal squamous cell carcinoma, laryngeal squamous cell hyperplasia, radiomics, CT imaging, differential diagnosis

## Abstract

**Objective:**

To evaluate the clinical application of the CT-based radiomics prediction model for discriminating SCC and SCH.

**Methods:**

A total of 254 clinical samples were selected from 291 patients with larynx-occupying lesions who underwent primary surgery. All lesions were validated via histopathological examination at The Second Hospital of Jilin University between June 2004 and December 2019. All patients were randomly allocated to the training (*n* = 177) and validation (*n* = 77) cohorts. After the acquisition of CT images, manual 3D tumor segmentation was performed using the CT images of the arterial, venous, and non-contrast phases via ITK-SNAP software. Subsequently, radiomics features were extracted using A.K. software. Based on the above features, three different diagnostic models (CTN, CTA+CTV, and CTN+CTA+CTV) were constructed to classify squamous cell carcinoma (SCC) and squamous cell hyperplasia (SCH). Additionally, receiver operating characteristic (ROC) and decision curve analysis (DCA) curves were measured to evaluate the diagnostic characteristics and clinical safety of the proposed three prognostic models.

**Results:**

In the radiomic prediction Model 1 (CTN), the area under the curve (AUC), accuracy, sensitivity, specificity, positive predictive value (PPV) and negative predictive value (NPV) of the training cohorts in differentiating SCC and SCH were 0.883, 0.785, 0.645, 1.000, 1.000, and 0.648, while in the testing cohorts, these values were 0.852, 0.792, 0.66, 1.000, 1.000, and 0.652, respectively. In the radiomic prediction Model 2 (CTA+CTV), the AUC, accuracy, sensitivity, specificity, PPV, and NPV values of the training cohorts were 0.965, 0.91, 0.916, 0.9, 0.933, and 0.875, respectively, while in the testing cohorts, the corresponding values were 0.902, 0.805, 0.851, 0.733, 0.833, and 0.759, respectively. In the radiomic prediction Model 3(CTN+CTA+CTV), the AUC, accuracy, sensitivity, specificity, PPV, and NPV values of the training cohorts were 0.985, 0.944, 0.953, 0.929, 0.953, and 0.929, while in the testing cohorts, the corresponding values were 0.965, 0.857, 0.894, 0.8, 0.875, and 0.828, respectively.

**Conclusion:**

The radiomic prediction Model 3, based on the arterial-venous-plain combined scan phase of CT, achieved promising diagnostic performance, expected to be regarded as a preoperative imaging tool in classifying SCC and SCH to guide clinicians to develop individualized treatment programs.

## 1 Introduction

As the seventh most common cancer worldwide, squamous cell cancer of the head and neck (HNSCC) is a potential health concern worldwide with increased incidence and death rates. It mainly occurs in the lips, oral cavity, nasal cavity, paranasal sinuses, oropharynx, hypopharynx, larynx, and parotid gland (1). More than 500,000 individuals are diagnosed with HNSCC worldwide each year. Furthermore, it is estimated that the number of new cases is expected to exceed 1 million in 2020, with the number of deaths expected to exceed 500,000. Notably, Central Europe and Eastern Europe had the largest increased incidence among men over 55 years of age (2, 3). In one study, ~75% of head and neck cancers overall are caused by tobacco smoking and alcohol abuse, with the remaining other ~25% attributable to HPV infection (4, 5). Therefore, efforts to promote the HPV vaccination and reduce pharmacy tobacco sales could help reduce risk factors in patients with head and neck cancer (6, 7).

There are ~2,814,000 cancer deaths in China, and the incidence and mortality rates are steadily increasing. In almost all populations examined, the incidence of cancer is higher in men than women, and the mortality rate is almost equal to the morbidity rate (8, 9). Among all cancers, HNSCC occupies an important position which has seriously threatened the health and lives of the Chinese people (10). Another point is that the incidence of HNSCC is ~3 times more common in men than women. Therefore, this area of research to improve the early detection, early diagnosis, and early treatment of HNSCC is of high importance, which could possibly enable the patients to receive the best treatment within the shortest possible time to reduce injury and prevent death rates by improving their clinical treatment (11).

In recent years, radiomics has been applied in the medical field for medical diagnosis, treatment, and prognosis prediction (12, 13). Many of radiomics features can be extracted from regions of interest on medical images which can be associated with clinical diagnosis and biological characteristics to build a diagnostic model, thereby improving the diagnostic efficacy (14). Integration of big data and medicine has raised new hopes for personalized medicine, and radiomics has become more feasible, extracting large amounts of data from medical images. CT has the characteristics of quick scanning speed and high repeatability, making it the most preferred inspection method to detect early HNSCC. Conventional examination methods are easily susceptible to the influence of a physician's subjective experiences. Furthermore, morphological characteristics supplied by CT are insufficient to evaluate the biological characteristics of the primary tumor. At present, radiomics overcomes the insufficiency of the above traditional imaging techniques, widely used in clinical diagnosis, treatment, and prognosis (15, 16). The qualitative and quantitative assessment of lesion characteristics and intratumoral spatial heterogeneity can contribute to improving the non-invasive preoperative diagnosis accuracy of HNSCC. This approach also provides personalized adjuvant treatment programs (17). What has been inquired into in this research is the evaluation of the clinical application of a CT-based radiomics prediction model for discriminating laryngeal squamous cell carcinoma (SCC) and squamous cell hyperplasia (SCH).

## 2 Materials and methods

### 2.1 Patient characteristics

**Figure 1:**
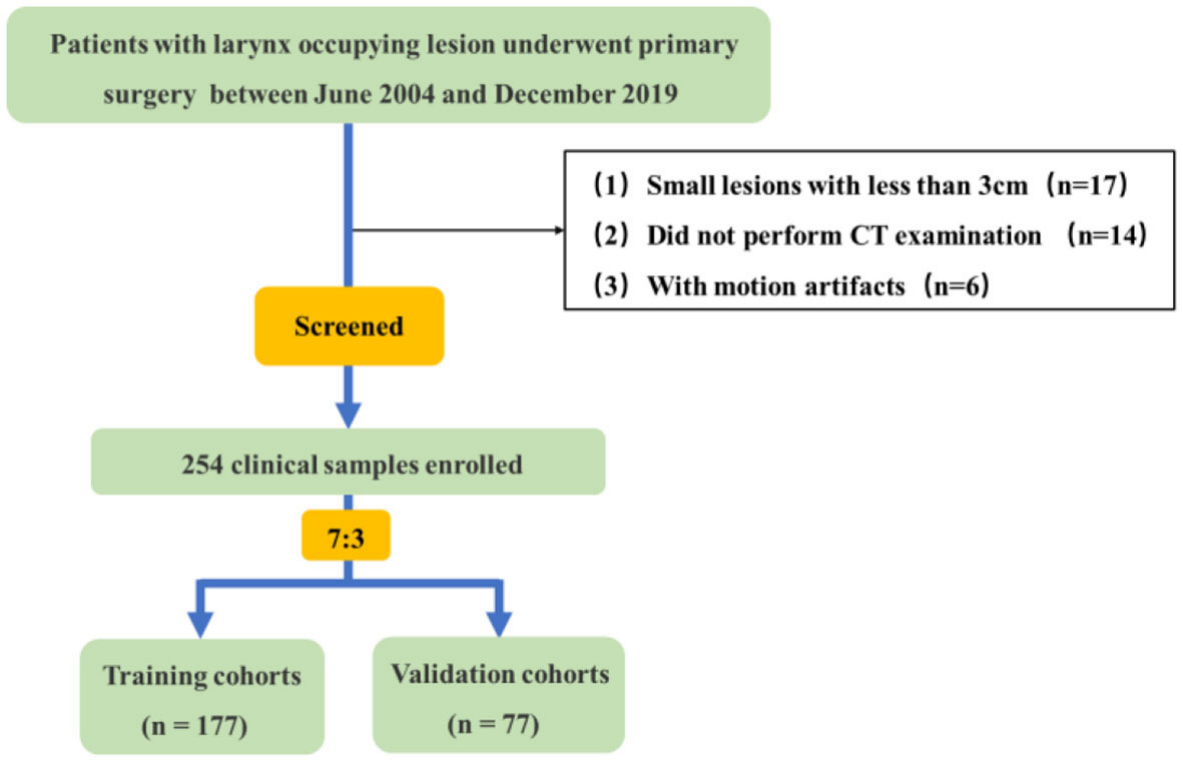
Flowchart of the patient selection process.

In this retrospective study, a total of 254 clinical samples were selected from 291 patients with larynx-occupying lesions who underwent primary surgery, and all lesions were validated via histopathological examination at The Second Hospital of Jilin University between June 2004 and December 2019. A flowchart is presented in Figure 1 to represent the process of selecting patients. All patients underwent preoperative unenhanced and dual-phase contrast-enhanced CT examination of the neck. Clinical-pathological data including age, gender, smoking and drinking history, pathological grade, tumor size, and clinical stage were collected. They were divided into two groups: the first group of patients were diagnosed with SCC while the second group consisted of those diagnosed with SCH. The study involved 227 men and 27 women with an average age of 44–85 years. Among them, 209 patients were smokers while 45 patients were non-smokers, and 202 patients reported alcohol consumption, while 52 patients did not drink.

The study included the following inclusion criteria: (a) Patients with primary larynx-occupying lesion; (b) No medical history of preoperative chemoradiotherapy; (c) Complete clinical and pathological diagnosis data were acquired; (d) Plain scan plus conventional dual-phase enhanced scan (arterial phase and venous phase) were implemented before the operation with complete image information. The exclusion criteria were as follows: (a) The lesions measured 10mm or less in diameter, partially becoming superficial; (b) No neck CT examination before the operation; (c) Low-quality CT images due to movement or artifacts.

### 2.2 CT radiomics analysis

**Figure 2:**
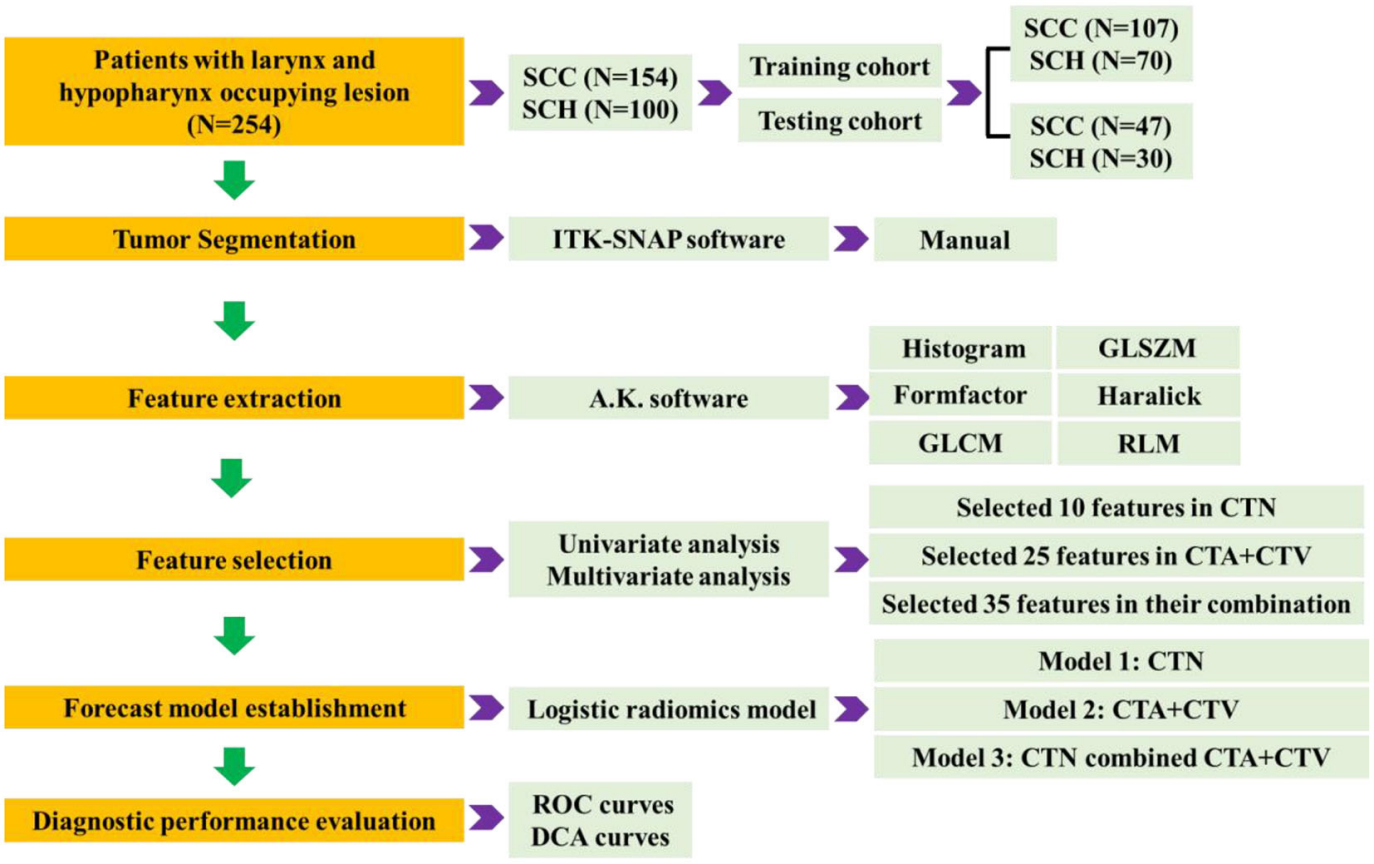
Graphical summarization of radiomics models analysis process.

The radiomics analysis process mainly includes five phases: CT image acquisition, ROI segmentation, feature extraction, feature selection, forecast model establishment, and diagnostic performance evaluation, as shown in Figure 2.

#### 2.2.1 CT image acquisition

All patients underwent a neck CT (iCT 256, Philips, Netherlands) to collect plain scan plus conventional dual-phase enhanced scans (arterial phase and venous phase). In general, the scanning neck range was from the inferior margin of the foramen magnum to the upper edge of the aortic arch, in which the patients were laid in the supine position with the neck completely exposed during the scanning. The most important parameters for the neck tissue CT scan requested here are as follows: the tube voltage 120kVp, electric current of 200mAs, scan time raging from 1s to 3s, slice thickness of 1 mm, matrix size of 256 × 256, and pitch ratio of 0.342. For an enhanced scan, iodinated contrast material was intravenously injected at a dose of 1.5 mL/kg, with an injection rate of 3.5 mL/s. The time for the arterial phase scan after injection was 35 s, whereas the time for the portal venous scan was 65 s.

#### 2.2.2 ROI segmentation

Three-dimensional manual segmentation of tumor in axial CT image of plain, arterial, and venous phase utilized via ITK-SNAP software (v.3.8.0; www.itksnap.org). Under the random and double-blind method, the boundaries of each layer were manually delineated on tumors by two radiologists with head and neck CT imaging diagnostic experience, and the region of interest (ROI) was composited. The tumor ROI segmentation in the larynx and hypopharynx was performed by a junior radiologist (with 5 years of experience) and reviewed by another senior radiologist (with 10 years of experience). In case of occurrence of a dispute, the final decision was made after the discussion between two doctors.

#### 2.2.3 Feature extraction

High-throughput quantitative features are extracted from 3D ROI of tumor lesions in three-phasic CT scan, from which mainly include histogram, gray-level size zone matrix (GLSZM), formfactor, haralick, gray-level cooccurrence matrix (GLCM), run length matrix (RLM) by A.K. software (Analysis Kit, GE Healthcare). In total, 1,188 quantitative radiomics features were extracted for each patient, with 396 features from each of the plain, arterial, and venous phases, respectively.

#### 2.2.4 Feature selection and forecast model establishment

We implement a comprehensive feature selection to establish the final forecast model via IPM statistics (V1.1.463 GE Healthcare, Shanghai, China). Before feature selection, the collected 1188 quantitative radiomics features were preprocessed, and feature normalization were employed. We first proposed that variables with zero variance were excluded from analyses. Then, the missing values and outlier values were replaced by the median. Finally, the data were standardized by standardization.

The 254 tumors will be randomly allocated based on a 7:3 ratio between the training cohort and the testing cohort, where 177 patients were used as the training cohort for feature selection and model building. The remaining 23 patients were regarded as the testing cohort for verifying the selected features and forecast model. Feature selection was implemented employing univariate (using Variance, Correlation_xx, and General_Univariate_analysis) and multivariate analyses (using L1) with stepwise selection-based dimensionality reduction algorithm for all the features. To further avoid model overfitting, 10-foldcross-validation was performed to recurve the selection of redundancy features and the least absolute shrinkage and selection operator (LASSO) regression to effectively eliminate a sequence of regression coefficients to exactly zero. Furthermore, a set of optimal features, which were compared by the Wilcoxon test, were obtained.

Subsequently, Model 1 (CTN) was constructed based on 10 optimal features; Model 2 (CTA+CTV) was constructed based on 25 optimal features; and Model 3 (CTN+CTA+CTV) considered all 35 parameters, extracted in the combination of Model 1 and Model 2 using logistic regression for discriminating laryngeal SCC and SCH. The differential diagnostic effectiveness and performance of the proposed three models were measured by the receiver operating characteristic (ROC) curves, area under the curve (AUC), sensitivity, specificity, positive predictive value (PPV), and negative predictive value (NPV). The calibration curves were measured to analyze the goodness of fit of the prediction models. In addition, the decision curve analysis (DCA) was adapted to evaluate the clinical efficacy and safety of the three models.

### 2.3 Statistical analysis

All statistical analyses were conducted based on IPM statistics. The continuous variables were assessed by the Mann–Whitney *U-*test or independent samples *T-*test, and the categorical variables were investigated using the chi-squared or Fisher exact tests. A two-tailed test with a *p* < 0.05 (typically ≤ 0.05) was indicative of a statistically significant difference.

## 3 Results

### 3.1 Clinical characteristics of the patients

Table 1 shows the characteristics of the 254 patients, with 154 patients diagnosed with SCC and 100 patients diagnosed with SCH. Statistical analysis for clinical data, such as age, gender, smoking status, alcohol consumption, and tumor location described above, was performed. The comparison of the two groups yielded a *p* > 0.05, revealing that there were similar between SCC and SCH in the training and testing cohorts.

**Table 1:** The risk factor analysis of larynx-occupying lesions in the training and testing cohorts examining clinical characteristics.

	**Training cohort**			**Testing cohort**			***P*-value**
	**SCC (*****N*** = **107)**	**SCH (*****N*** = **70)**	***P*** **-value**	**SCC (*****N*** = **47)**	**SCH (*****N*** = **30)**	***P*** **-value**	
Age	61.9 (9.00)	60.0 (8.05)	0.150	60.2 (8. 27)	56.9 (6.90)	0.066	0.051
Sex			0.774			1.000	0.762
Female	11 (10.3%)	9 (12.9%)		4 (8.51%)	3 (10.0%)		
Male	96 (89.7%)	61 (87.1%)		43 (91.5%)	27 (90.0%)		
Smoking			0.656			0.643	0.067
No	17 (15.9%)	11 (15.7%)		4 (8.51%)	1 (3.33%)		
Yes	90 (84.1%)	59 (84.3%)		43 (91.5%)	29 (96.7%)		
Alcohol			0.759			0.186	0.651
No	20 (18.7%)	11 (15.7%)		9 (19.1%)	2 (6.67%)		
Yes	87 (81.3%)	59 (84.3%)		38 (80.9%)	28 (93.3%)		
Tumor location			0.476			0.951	0.603
Supraglottis	27 (25.27%)	19 (27.1%)		11 (23.43%)	6 (20.0%)		
Glottis	68 (63.6%)	47 (67.1%)		33 (70.2%)	23 (76.7%)		
Subglottis	12 (11.2%)	4 (5.71%)		3 (6.38%)	1 (3.33%)		

### 3.2 Radiomic feature selection and model building

**Figure 3:**
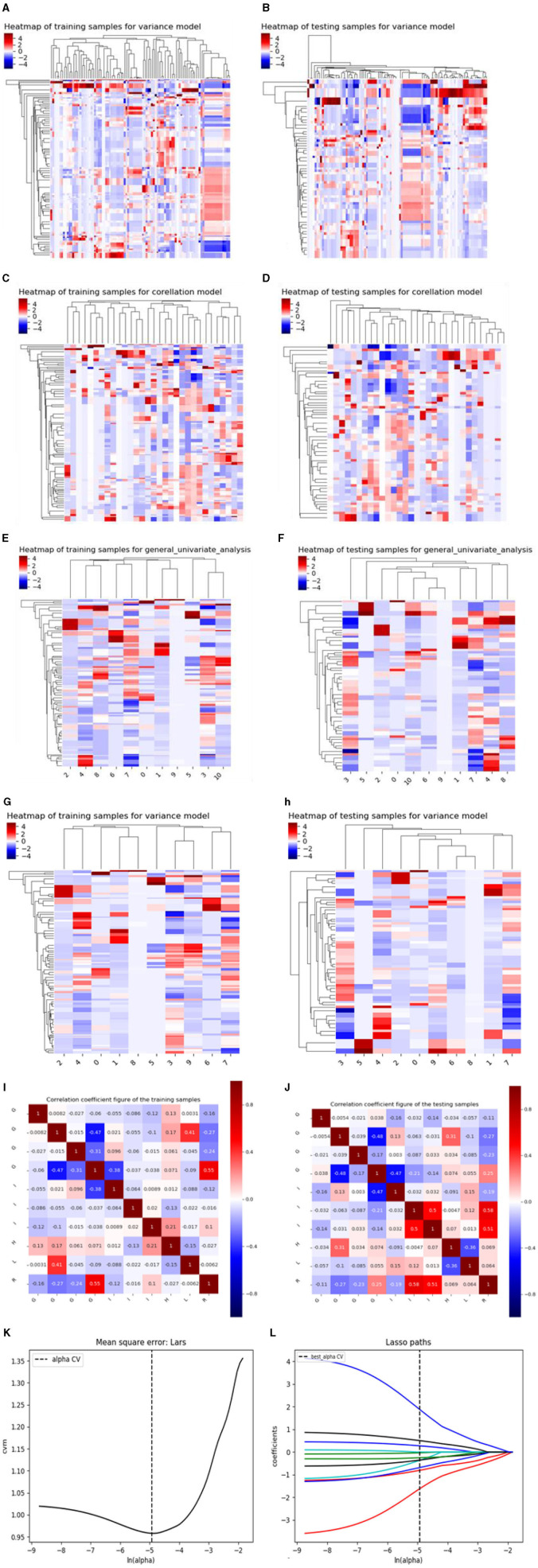
**(A–J)** shows the correlation heat maps of Model 1 demonstrating correlations between features in the training and testing cohort. **(K, L)** shows the results of the 10-fold cross-validation method and LASSO regression analysis, respectively, removing highly redundant features to obtain the optimal features.

There are three steps in building Model 1: For the CT plain scan, a total of 396 features were first subjected to the variance method (threshold: 1.0) to screen out 98 features. Next, we adopted the procedure of Correlation_xx method with a cutoff set to 0.7 to remove redundant features, resulting in remaining 31 parameters. Then, a total of 11 features were retained via General_Univariate_analysis (*p*-value threshold 0.05). Finally, the remaining 10 features were obtained using the L1 method, revealing an obvious difference between SCC and SCH in the training cohorts. A correlation heat map (Figures 3A–J) revealed that strong positive correlation radiomics features were sufficient to receive an obviously differential diagnosis. Figure 3K shows the results of the tenfold cross-validation method, and Figure 3L shows the results of the LASSO regression analysis. Subsequently, 10 features from the CT plain scan were finally selected, and the optimal parameters are as follows: [“GLCMEnergy_angle0_offset7”], [“GLCMEnergy_angle90_offset4”], [“GLCMEnergy_angle90_offset7”], [“GLCMEntropy_angle90_offset1”], [“InverseDifferenceMoment_AllDirection_offset1_SD”], [“InverseDifferenceMoment_AllDirection_offset7_SD”], [“InverseDifferenceMoment_angle135_offset7”], [“HighGreyLevelRunEmphasis_angle90_offset1”], [“LongRunLowGreyLevelEmphasis_AllDirection_offset4_SD”], [“RunLengthNonuniformity_angle90_offset7”].

Based on above single factor analysis and multifactor analysis, a total of 10 most predictive features and coefficients for constructing the optimal radiomics signatures Model 1(Figure 5A). Consequently, rad-score was calculated by selected 10 features weighted as below: Rad-score (Model 1)=0.9334 + 0.4575[“GLCMEnergy_angle0_offset7”]-1.2571[“GLCMEnergy_angle90_offset4”]-0.0960[“GLCMEnergy_angle90_offset7”]-1.1963[“GLCMEntropy_angle90_offset1”]-0.6162[“InverseDifferenceMoment_AllDirection_offset1_SD”]-1.3213[“InverseDifferenceMoment_AllDirection_offset7_SD”]-3.6916[“InverseDifferenceMoment_angle135_offset7”]-0.2776[“HighGreyLevelRunEmphasis_angle90_offset1”] +0.8819[“LongRunLowGreyLevelEmphasis_AllDirection_offset4 _SD”]+4.2529[“RunLengthNonuniformity_angle90_offset7”].

**Figure 4:**
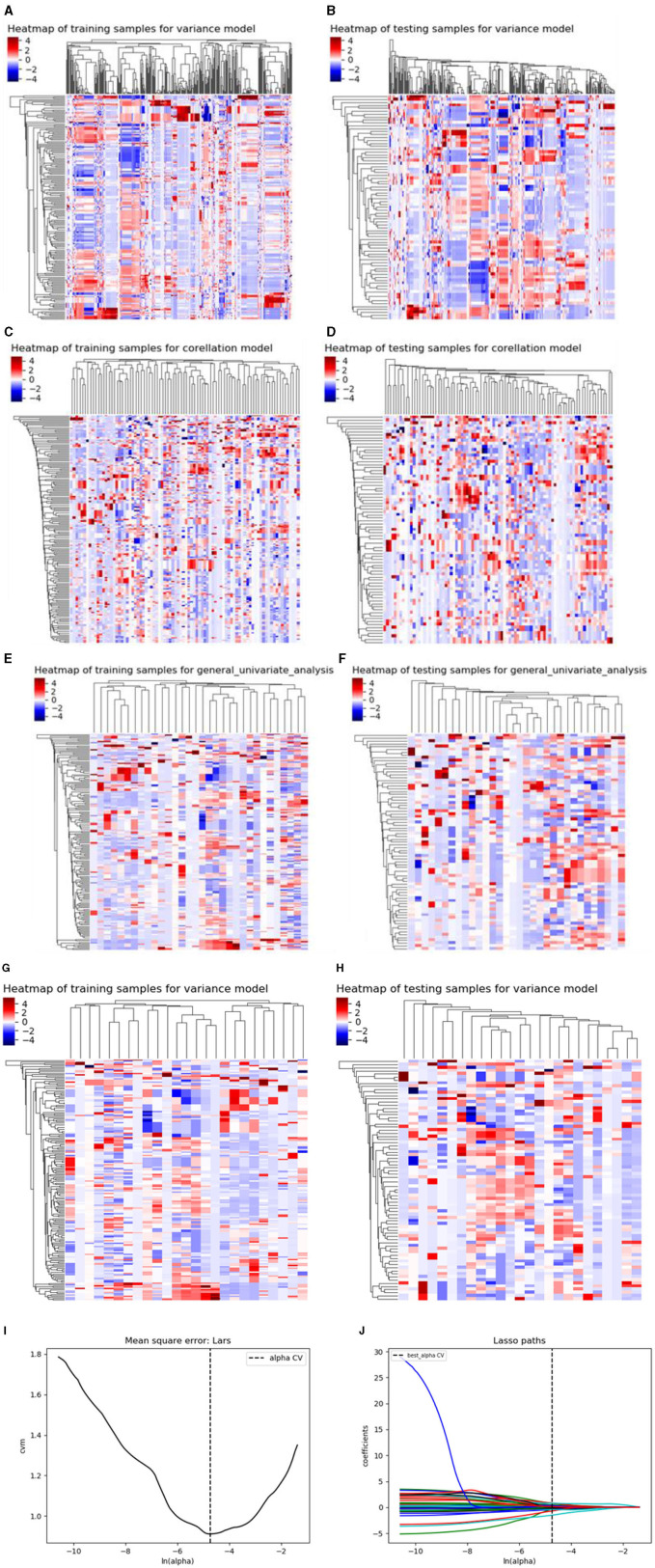
**(A–H)** shows the correlation heat maps of Model 2 demonstrating the correlations between features in the training and testing cohort. **(I, J)** show the results of the 10-fold cross-validation method and LASSO regression analysis, respectively, removing highly redundant features to obtain the optimal features.

Using similar feature reduction methods, 25 features from the CT conventional dual-phase enhanced scan were finally selected by specific methods listed in four steps: In the first step, after applying the variance method (threshold: 1.0), the 792 features extracted by the conventional dual-phase enhanced scan were reduced to 331 features. Next, we obtained a feature count of 86 parameters by the Correlation_xx method (cutoff: 0.7). Then, General_Univariate_analysis was utilized to remove 54 features, and 32 radiomics features remained. Finally, the remaining 25 features were obtained using the L1 method. A correlation heat map (Figures 4A–H) revealed that strong positive correlation radiomics features were sufficient to receive an obviously differential diagnosis. Figure 3I shows the results of the tenfold cross-validation method, and Figure 3J shows the results of the LASSO regression analysis. The optimal parameters are as follows: [“V_ClusterProminence_angle0_offset7”], [“V_Correlation_angle0_offset4”], [“V_GLCMEnergy_angle90_offset7”], [“V_HaralickCorrelation_angle45_offset7”], [“V_Inertia_AllDirection_offset4_SD”], [“V_Inertia_AllDirection_offset7”], [“V_InverseDifferenceMoment_angle0_offset7”], [“V_GreyLevelNonuniformity_AllDirection_offset4_SD”], [“V_Compactness2”], [“V_Maximum3DDiameter”], [“A_ClusterProminence_angle90_offset7”], [“A_ClusterShade_angle135_offset1”], [“A_ClusterShade_angle45_offset4”], [“A_ClusterShade_angle90_offset1”], [“A_ClusterShade_angle90_offset7”], [“A_GLCMEnergy_angle90_offset4”], [“A_GLCMEntropy_angle45_offset4”], [“A_HaralickCorrelation_angle135_offset4”], [“A_HaralickCorrelation_angle90_offset7”], [“A_Inertia_AllDirection_offset1_SD”], [“A_HighGreyLevelRunEmphasis_AllDirection_offset7_SD”], [“A_LongRunLowGreyLevelEmphasis_angle45_offset7”], [“A_RunLengthNonuniformity_AllDirection_offset1”], [“A_RunLengthNonuniformity_AllDirection_offset7_SD”], [“A_ShortRunHighGreyLevelEmphasis_AllDirection_offset1_SD”].

**Figure 5:**
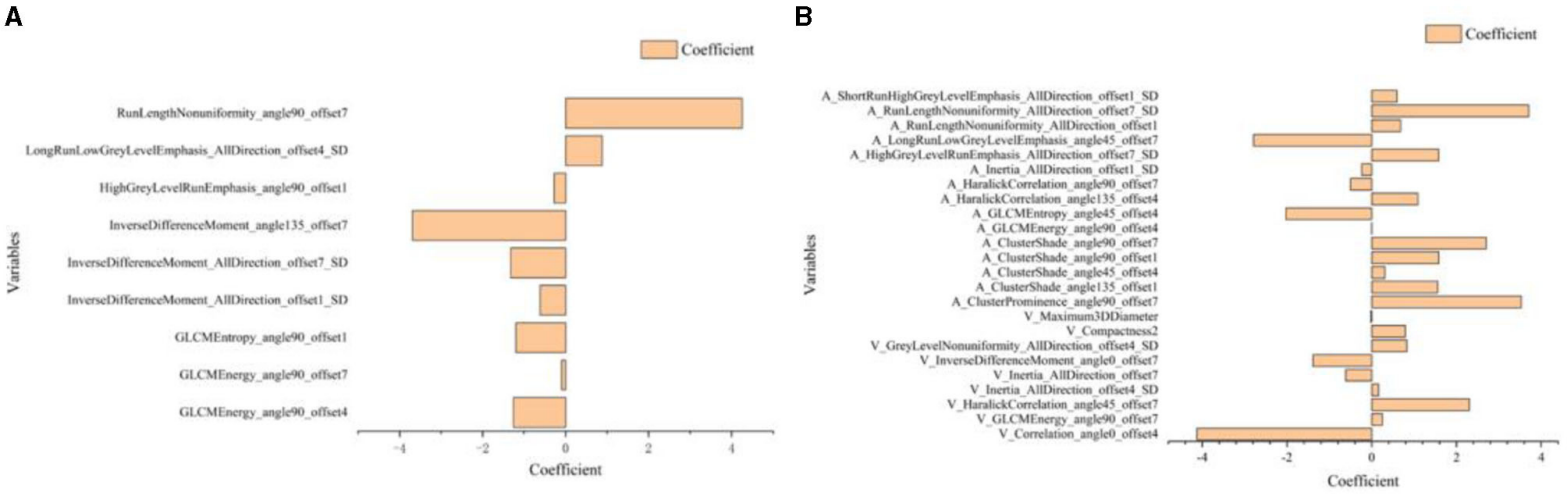
Histogram shows the 10 most predictive radiomics features obtained in Model 1 **(A)** and 25 most predictive radiomics features obtained in Model 2 **(B)**.

Based on the above single-factor analysis and multifactor analysis, a total of 25 most predictive features and coefficients were identified for constructing the optimal radiomics signatures in Model 2 (Figure 5B). The Rad-score was calculated using the selected 25 features weighted as below: Rad-score (Model 2) = 3.1869 + 1.4625[“V_ClusterProminence_angle0_offset7”]-4.1270[“V_Correlation_angle0_offset4”]+0.2540[“V_GLCMEnergy_angle90_offset7”]+2.3007[“V_HaralickCorrelation_angle45_offset7”]+0.1652[“V_Inertia_AllDirection_offset4_SD”]-0.6157[“V_Inertia_AllDirection_offset7”]-1.3833[“V_InverseDifferenceMoment_angle0_offset7”]+0.8270[“V_GreyLevelNonuniformity_AllDirection_offset4_SD”]+0.7914[“V_Compactness2”]-0.0179[“V_Maximum3DDiameter”]+3.5277[“A_ClusterProminence_angle90_offset7”]+1.5561[A_ClusterShade_angle135_offset1”']+0.3104[“A_ClusterShade_angle45_offset4”]+1.5868[“A_ClusterShade_angle90_offset1”]+2.7000[“A_ClusterShade_angle90_offset7”]-0.0034[“A_GLCMEnergy_angle90_offset4”]-2.0308[“A_GLCMEntropy_angle45_offset4”] +1.0889[“A_HaralickCorrelation_angle135_offset4”]-0.4997[“A_HaralickCorrelation_angle90_offset7”] -0.2386[“A_Inertia_AllDirection_offset1_SD”] +1.5807[“A_HighGreyLevelRunEmphasis_AllDirection_offset7_SD”]-2.7901[“A_LongRunLowGreyLevelEmphasis_angle45_offset7”]+0.6788[“A_RunLengthNonuniformity_AllDirection_offset1”]+3.7087[“A_RunLengthNonuniformity_AllDirection_offset7_SD”]+0.5963[“A_ShortRunHighGreyLevelEmphasis_AllDirection_offset1_SD”]

**Figure 6:**
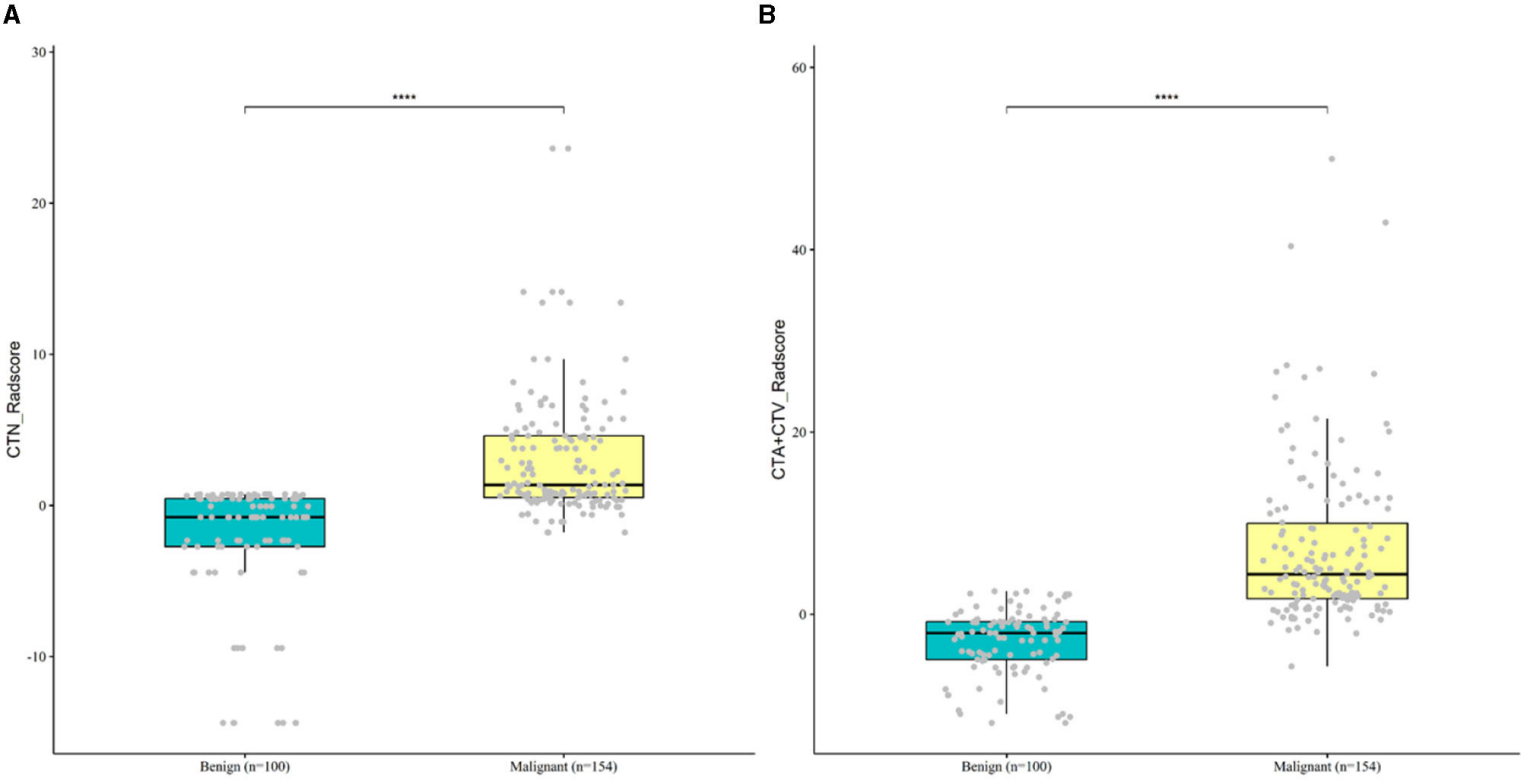
Boxplot of differentially Rad-score for malignant and benign case in Model 1 **(A)** and Model 2 **(B)**.

To comprehensively and intuitively demonstrate the characteristics and differences of the dataset, we put together the boxplots of radiomics scores for benign and malignant cases. Based on all collected clinical dataset, Figure 6 shows that the Rad-score was significantly upregulated in malignant patients as compared to benign controls in both Model 1(CTN) and Model 2(CTAV).

As shown in Table 2, the Wilcoxon rank sum test was used to compare the Rad-scores of Model 1 and Model 2 for discriminating SCC and SCH in both the training and testing cohorts (both *P* < 0.001).

**Table 2:** The Wilcoxon rank sum test of Rad-scores in Model 1 and Model 2 for training and testing cohorts.

	**Training cohort (*P*-value)**	**Validation cohort (*P*-value)**
Model 1	< 0.001^*^	< 0.001^*^
Model 2	< 0.001^*^	< 0.001^*^

^*^Indicates that the difference is significant.

The process of constructing the three-period combined Model 3 is presented as follows: The 10 optimal radiomics signatures selected from Model 1 and the 25 optimal radiomics signatures from Model 2 were considered, incorporating all 35 parameters to establish Model 3 (CTN+CTA+CTV) using logistic regression for discriminating laryngeal SCC and SCH.

### 3.3 Comparing the performance of the three different models

The corresponding performance evaluation criteria for differentiating SCC and SCH contained AUC, accuracy, sensitivity, specificity, PPV, and NPV for each model. In radiomic prediction Model 1(CTN), the measured values of the training cohort were 0.883, 0.785, 0.645, 1.000, 1.000, and 0.648, while in the testing cohorts were 0.852, 0.792, 0.66, 1.000, 1.000, and 0.652. In radiomic prediction Model 2 (CTA+CTV), the measured values of training cohorts were 0.965, 0.91, 0.916, 0.9, 0.933, and 0.875, while in the testing cohorts were 0.902, 0.805, 0.851, 0.733, 0.833, and 0.759. In radiomic prediction Model 3 (CTN+CTA+CTV), the measured values of training cohorts were 0.985, 0.944, 0.953, 0.929, 0.953, and 0.929, while in the testing cohorts were 0.965, 0.857, 0.894, 0.8, 0.875, and 0.828, respectively. Among them, Model 3 has the highest performance and can be used for predicting differential clinical diagnosis.

**Figure 7:**
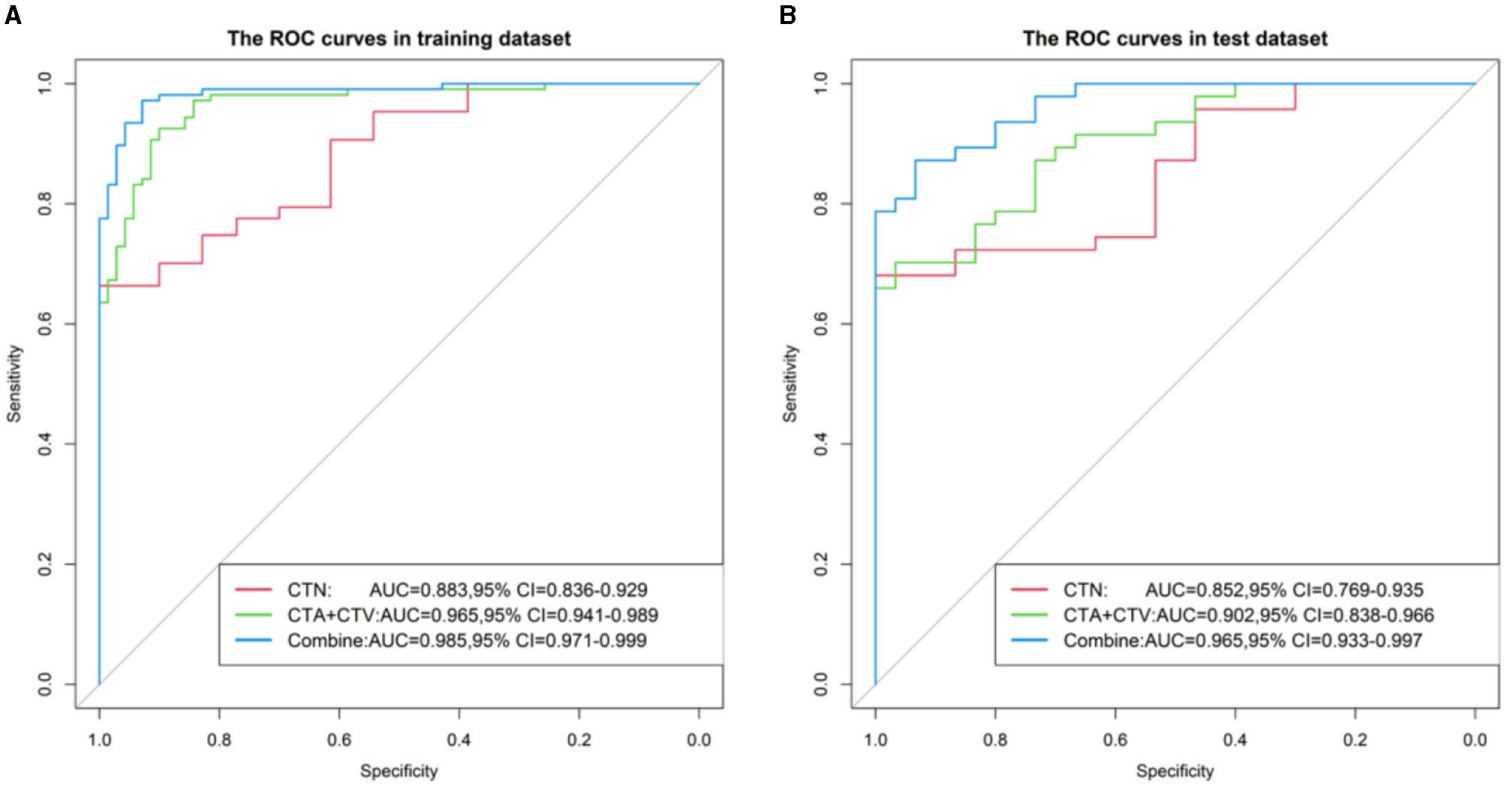
ROC curve of three Models (CTN, CTA+CTV, and CTN+CTA+CTV) in the training **(A)** and testing cohorts **(B)**.

**Figure 8:**
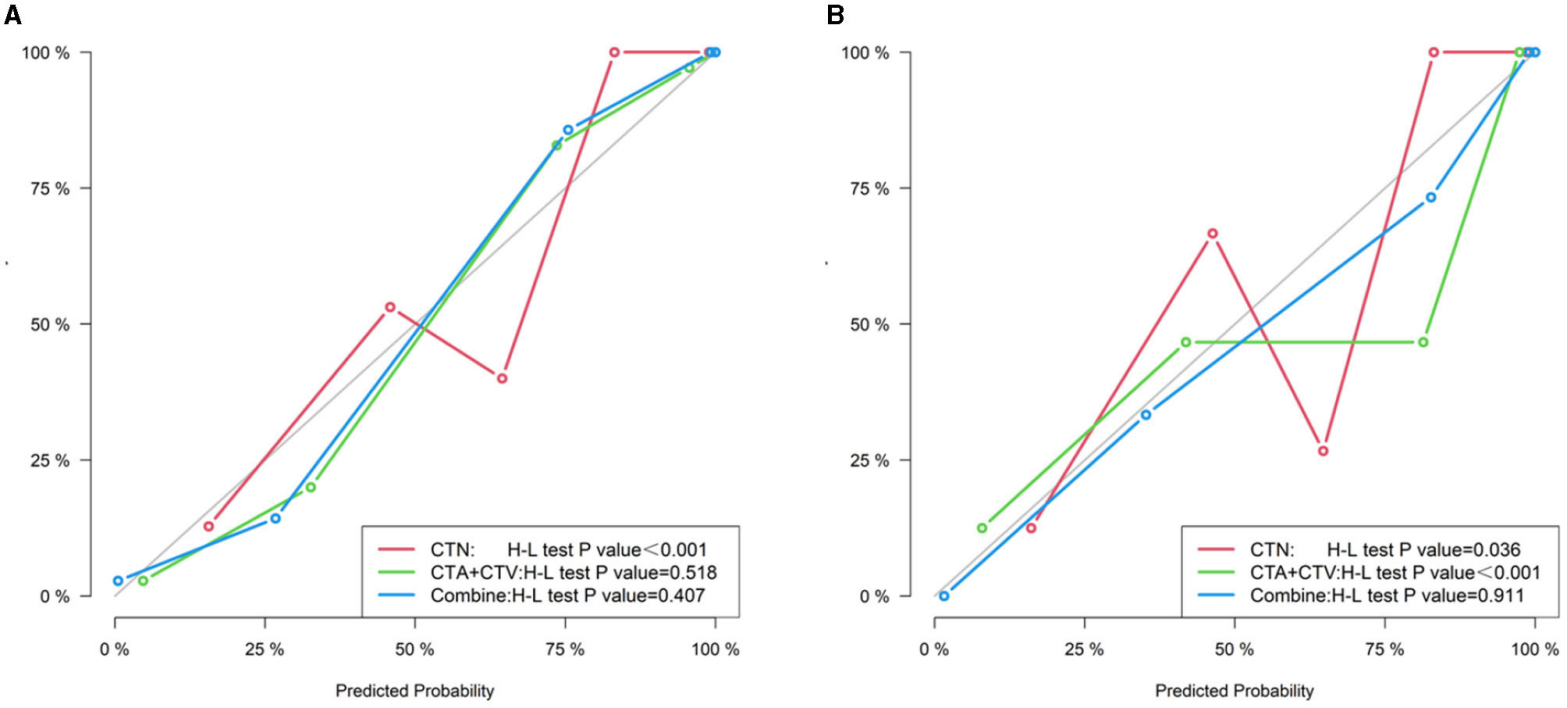
Calibration curve of three models (CTN, CTA+CTV, and CTN+CTA+CTV) in the training **(A)** and testing cohorts **(B)**.

We constructed the calibration curve to describe the degree of calibration of the three models in the training (Figure 7A) and testing (Figure 7B) cohorts, which illustrates that the closer the calibration curves (red, green and blue) are to the standard curve (black), the better the calibration capability of the model. As shown in Figure 8, the actual prediction performance of the prediction Model 3 has a good consistency.

**Figure 9:**
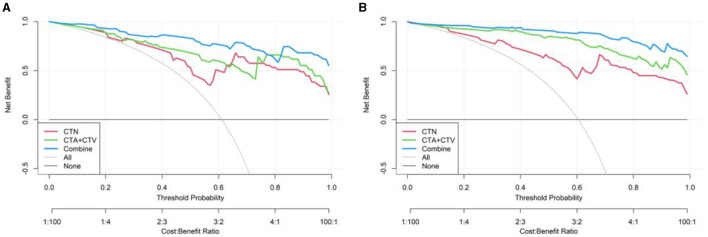
Decision curve of three models (CTN, CTA+CTV, and CTN+CTA+CTV) in the training **(A)** and testing cohorts **(B)**.

We also established a calibration curve with a DCA curve for evaluating three models in the training (Figure 9A) and testing (Figure 9B) cohorts, which indicated that a larger area under the decision curve indicated a better clinical practicability. As shown in Figure 9, within the safe range, the DCA indicated that the net benefit of the prediction was higher for all three models, with Model 3, exhibiting the highest net benefit, and the benefit rate of the population would reach its maximum.

## 4 Discussion and conclusion

Radiomics is an emerging field of image analysis with potential applications for diagnosis, treatment response, and prognosis evaluation in cancer patients via computer-aided diagnosis (CAD) technology, which solved many clinical problems, reduced misdiagnosis rate, and decreased the radiologist workload (18). In our study, radiomics used computational algorithms to convert medical imaging information into high-resolution quantitative mineable “big data” and aimed to further build a more reliable predictive and differential diagnosis model (19).

A multicenter study by Zhang et al. (20) showed that different machines and different CT scanning parameters might influence the radiomics result. Therefore, CT image data extraction is the basis of radiomics. It is important to note that CT scanning parameters could be consistent for radiomics analysis, in which the CT images were collected uniformly. We were doubtful that different CT scanning parameters might affect the radiomics optimal features and prediction model establishment.

The radiomics features of the entire primary larynx-occupying lesion were extracted from the CT images of the arterial, venous, and non-contrast phases, which have a positive effect on establishing a differential diagnosis model for discriminating SCC and SCH. We concluded that the AUC, accuracy, sensitivity, specificity, PPV, and NPV of the optimal Model 3 in training cohorts for differentiating SCC and SCH were 0.985, 0.944, 0.953, 0.929, 0.953, and 0.929, respectively, while in the testing cohorts were 0.965, 0.857, 0.894, 0.8, 0.875, and 0.828, respectively. Based on the research results, a comprehensive comparative analysis of the consequences of these three models revealed that the amalgamation of characteristic parameters from the plain-arterial-venous combined model provided more optimal radiomics parameters than from the plain model or arterial-venous combined model alone. Thus, Model 3 based on arterial-venous-plain combined scan phase of CT has good discriminative performance with high sensitivity and specificity in SCC and SCH.

Additionally, we collected related important clinical characteristics of all patients, including age, gender, smoking status, and alcohol consumption. Among them, smoking status and alcohol consumption were the major risk factors leading to the high incidence of laryngeal cancer. Among the enrolled patients, 209 patients had a smoking history and 45 patients had no smoking history. We analyzed that long-term smokers are more susceptible to developing laryngeal cancer, taking into account consistent smoking initiation and current smoking status. In terms of alcohol consumption, 202 patients had a drinking history and 52 patients have no drinking history. Similarly, we found that long-term drinkers are more susceptible to developing laryngeal cancer, taking into account the drinking start times and current drinking quantity. Therefore, clinicians will be able to advise their patients to quit smoking and drinking, thereby extending overall patient survival time (21, 22).

Chen et al. (23) evaluated the use of venous-phase CT images to develop a radiomics model, a deep learning model, and a combined model to predict preoperative staging in stratifying patients with laryngeal carcinoma. The authors demonstrated that the combined model performed significantly capability than a radiologist in stratifying patients into stage I–II and stage III–IV. The AUCs, which indicated model diagnostic performance, assessed the accuracy of a model. The radiomics model, DL model, and combined model for distinguishing staging ability in the test set were 0.704 (95% CI: 0.588–0.820), 0.724 (95% CI: 0.613–0.835), and 0.849 (95% CI: 0.755–0.943), respectively. This study confirmed the application value of radiomics in accurate preoperative staging of laryngeal cancer. Kang et al. (24) developed a radiomics nomogram to analyze 114 patients with advanced laryngeal cancer after induction chemotherapy. The experiments demonstrated that the Rad-score was an independent predictor. In addition, clinical factors were incorporated to build radiomics nomogram which predicted the pathological response and overall survival. This study proved that CT radiomics nomogram possesses the best predictive property in the pathological response after induced chemotherapy and overall survival. Therefore, we fused plain scan and conventional dual-phase enhanced scanning to the radiomics model to improve the predictive performance.

While the radiomics models are promising in clinical practice, there are several key limitations to this study that require attention. First, the model was established based on the single-center nature of the research that lacked prospective multicenter external validation of our findings. Second, we only collected the CT examination images; however, not collect MRI data, which may cause potential data bias. Finally, the number of clinical samples size is relatively limited, which expanded sample size to further research.

To the best of our knowledge, with the supplement of related important clinical characteristics, Model 3 based on arterial-venous-plain combined scan phase of CT has important clinical significance in distinguishing SCC from SCH. In conclusion, our results demonstrated that radiomics could provide valuable information and play an important role in preoperative diagnosis and clinical treatment to guide clinicians to develop individualized treatment programs (25, 26).

## Data availability statement

The datasets presented in this article are not readily available because protect the privacy of enrolled patient information. Requests to access the datasets should be directed to FC, 574267524@qq.com.

## Ethics statement

The studies involving humans were approved by the Ethics Committee of Second Hospital of Jilin University. The studies were conducted in accordance with the local legislation and institutional requirements. Written informed consent for participation was not required from the participants or the participants' legal guardians/next of kin because Since this study was retrospective, it was not necessary to obtain written informed consent from patients. Written informed consent was not obtained from the individual(s) for the publication of any potentially identifiable images or data included in this article because Since this study was retrospective, it was not necessary to obtain written informed consent from patients.

## Author contributions

FC: Writing—original draft. OK: Writing—review & editing. WL: Writing—review & editing. JL: Writing—review & editing. QY: Writing—review & editing.
